# *Prunus* genetics and applications after de novo genome sequencing: achievements and prospects

**DOI:** 10.1038/s41438-019-0140-8

**Published:** 2019-04-05

**Authors:** Maria José Aranzana, Véronique Decroocq, Elisabeth Dirlewanger, Iban Eduardo, Zhong Shan Gao, Ksenija Gasic, Amy Iezzoni, Sook Jung, Cameron Peace, Humberto Prieto, Ryutaro Tao, Ignazio Verde, Albert G. Abbott, Pere Arús

**Affiliations:** 1 IRTA, Centre de Recerca en Agrigenòmica CSIC-IRTA-UAB-UB, Campus UAB, Edifici CRAG, Cerdanyola del Vallès (Bellaterra), 08193 Barcelona, Spain; 2 UMR 1332 BFP, INRA, University of Bordeaux, A3C and Virology Teams, 33882 Villenave-d’Ornon Cedex, France; 3 Allergy Research Center, Zhejiang University, 310058 Hangzhou, China; 4 Clemson University, Clemson, SC 29634 USA; 5 Department of Horticulture, Michigan State University, 1066 Bogue Street, East Lansing, MI 48824-1325 USA; 6 Department of Horticulture, Washington State University, Pullman, WA 99164-6414 USA; 7 Biotechnology Laboratory, La Platina Research Station, Instituto de Investigaciones Agropecuarias, Santa Rosa, 11610 La Pintana, Santiago Chile; 8 Laboratory of Pomology, Graduate School of Agriculture, Kyoto University, Kyoto, 606-8502 Japan; 9 Consiglio per la ricerca in agricoltura e l’analisi dell’economia agraria (CREA) – Centro di ricerca Olivicoltura, Frutticoltura e Agrumicoltura (CREA-OFA), Rome, Italy; 10 University of Kentucky, 106 T. P. Cooper Hall, Lexington, KY 40546-0073 USA

**Keywords:** Plant breeding, Genomics, Agricultural genetics

## Abstract

Prior to the availability of whole-genome sequences, our understanding of the structural and functional aspects of *Prunus* tree genomes was limited mostly to molecular genetic mapping of important traits and development of EST resources. With public release of the peach genome and others that followed, significant advances in our knowledge of *Prunus* genomes and the genetic underpinnings of important traits ensued. In this review, we highlight key achievements in *Prunus* genetics and breeding driven by the availability of these whole-genome sequences. Within the structural and evolutionary contexts, we summarize: (1) the current status of *Prunus* whole-genome sequences; (2) preliminary and ongoing work on the sequence structure and diversity of the genomes; (3) the analyses of *Prunus* genome evolution driven by natural and man-made selection; and (4) provide insight into haploblocking genomes as a means to define genome-scale patterns of evolution that can be leveraged for trait selection in pedigree-based *Prunus* tree breeding programs worldwide. Functionally, we summarize recent and ongoing work that leverages whole-genome sequences to identify and characterize genes controlling 22 agronomically important *Prunus* traits. These include phenology, fruit quality, allergens, disease resistance, tree architecture, and self-incompatibility. Translationally, we explore the application of sequence-based marker-assisted breeding technologies and other sequence-guided biotechnological approaches for *Prunus* crop improvement. Finally, we present the current status of publically available *Prunus* genomics and genetics data housed mainly in the Genome Database for Rosaceae (GDR) and its updated functionalities for future bioinformatics-based *Prunus* genetics and genomics inquiry.

## A fruitful genomic resource

Detailed genomic maps from the fruit-bearing trees of the *Prunus* genus should greatly accelerate the breeding of superior cultivars. These plants produce a variety of popular fruits, but until relatively recently, breeders had only limited genetic information with which to work. Pere Arús of Spain’s Institute of Agrifood Research and Technology and colleagues have now reviewed the rapid advances in *Prunus* genomics that have taken place since 2010, when researchers completed the first genome assembly for this genus. In the ensuing decade, increasingly high-quality genomes from the peach, Japanese apricot, and cherry have allowed scientists to home in on chromosomal regions associated with key traits such as fruit quality and disease resistance. These data also offer valuable genetic markers that agronomists can use to guide the production of healthier trees and more commercially desirable fruits.

## Introduction

Stone fruit (peaches, cherries, apricots, plums) and almond belong to the *Prunus* genus, which encompasses approximately 250 species^1^ that share a small (250–300 Mbp) and highly syntenic genome^2,3^ with eight basic chromosomes. Most of the cultivated species are diploid with two exceptions, the hexaploid European plum (*P. domestica*) and the tetraploid tart cherry (*P. cerasus*). Sexual compatibility is frequent, particularly within members of the same subgenus: *Amygdalus* (peach-almond), *Prunus* (apricot-plum), and *Cerasus* (cherries), although certain crosses between species belonging to *Amygdalus* and *Prunus* subgenera are also possible.

The deployment of basic molecular technology during the past half century advanced our understanding of important aspects of *Prunus* genetics, including the consolidation of its phylogenetic relationships, the analysis of its genetic variability, the development of genetic markers and the construction and comparison of linkage maps, the integration of a large number of major genes and quantitative trait loci (QTLs) in these maps, and the identification of markers tightly linked to some traits enabling their selection in plant breeding^4^. Plausible hypotheses about candidate genes for certain characters were established, such as the genes responsible for the melting vs. nonmelting fruit flesh consistency (*M*/*m*) and freestone vs. clingstone flesh adherence to the stone (*F*/*f*)^5,6^, the gene for evergrowing (*Evg*/*evg*) for which the recessive homozygote is associated with continuous leaf growth and a failure to enter dormancy^7^, and the two genes involved in the self-incompatibility system^8^ (Table 1). However, positional cloning of trait-controlling genes was hampered by long intergeneration times, time and space constraints on progeny numbers in mapping crosses, marker availability in specific chromosomal regions, and finally by the lack of efficient transformation methods in most species other than hexaploid plum^9^.

**Table 1:** *Prunus* major genes cloned or fine mapped with strong candidate genes

Trait	Origin	Position^a^	Gene description^b^	Strategy	Causal polymorphism
Evergrowing	*P. persica*	G1 (*Evg*)	MADS box transcription factors/*DAM1-DAM6*	Mapping mutants, positional cloning, candidate gene (CG) functional classification and expression analysis^7^	Deletion in the DAM-box gene region of the *evg* mutant
Maturity date/slow ripening	*P. persica*	G4 (*MD*, *Sr*)	NAC transcription factor (*ppa008301m v1.0; Prupe.4G186800 v2.0)*	Fine mapping, whole-genome sequence analysis, and CG functional classification^48^/mapping, CG functional classification and cultivar genotyping^102^	In-frame insertion of 9 bp in the last exon for *MD*/gene deletion for *Sr*
Fruit size	*P. avium*	G2	Cell number regulator (*CNR*) (*PavCNR12)*	CG approach, segregation and association analysis^73^	–
Fruit size	*P. avium* *; P. persica*	G6	Cell number regulator (*PavCNR20; Prupe.6G240600)*	CG approach, segregation and association analysis^240^/pedigree-based QTL mapping^74^	–
Fruit shape	*P. persica*	G6 (*Sh*)	Leucine-rich repeat receptor-like kinase *(Prupe.6G281100)*	Linkage and association mapping, whole-genome sequencing, CG functional classification and expression analysis^79^	Large deletion affecting the promoter and part of the gene
Flesh color (white/yellow)	*P. persica*	G1 (*Y*)	Carotenoid cleavage dioxygenase (*PpCCD4; ppa006109; Prupe.1G255500*)	CG approach, whole-genome resequence analysis of cultivars and somatic mutants, gene expression and volatile compound analysis^81,82^	Three independent polymorphisms causing non-functional genes: 1 SNP, an SSR, and a transposable element (TE)
Flesh and skin color	*P. persica; P. avium; P. salicina*	G3 (*H*)	MYB transcription factor *(PavMYB10* *; PpMYB10.1)*	QTL analysis and CG functional classification^70,85–87^. Whole-genome sequence analysis^86^	SNPs in *P. avium*;^85^ INDEL in *P. persica*^86^
Blood flesh	*P. persica*	G5 (*DBF; Bl*)	NAC transcription factor *(ppa022238m; Prupe.5G006200)*	CG approach, comparative transcriptome and functional analysis^88^	–
Green vs. red leaf color	*P. persica*	G6 (*Gr*)	MYB transcription factor *(PpMYB10.4)*	Transcriptome comparison; transient expression in tobacco and peach leaves^241^	–
Fruit acidity	*P. persica*	G5 (*D*)	Auxin efflux carrier family protein (*ppa006339m; Prupe.5G004300*)	Fine mapping^93^, GWAS^28,94^, and expression analysis^28,121^	SNP
Melting vs. nonmelting flesh and clingston vs. freestone	*P. persica*	G4 (*M* and *F*)	Endopolygalacturonase *(Prupe.4G261900* and *Prupe.4G262200)*	Fine mapping and CG functional classification^5,6^	SNPs and small indels
Stony hard texture	*P. persica*	G6 (*Hd*)	*PpYUC11-like* (*ppa008176m*)	Fine mapping, GWAS, and CG approach^33,104^	TE
Hairy vs. glabrous skin	*P. persica*	G5 (*G*)	R2R3 MYB transcription factor (*PpeMYB25; ppa023143m; Prupe.5G196100*)	Fine mapping/whole-genome sequence analysis and CG functional classification^167^	TE
Root-knot nematode resistance	*P. cerasifera*	G7 (*Ma*)	Toll/interleukin1 receptor-nucleotide binding site-leucine-rich repeat (*TNL1*)	Fine mapping, positional cloning, and functional validation^131^	–
Brachytic dwarf	*P. persica*	G6 (*Dw*)	Giberellic acid (GA) receptor *(PpeGID1c; Prupe.6G332800)*	Sequencing‐based mapping strategy, sequence analysis, and functional validation in *P. domestica*^152^	Nonsense mutation
Weeping growth habit	*P. persica*	G3 (*Pl*)	SAM domain gene (*ppa013325; Prupe.3G200700*)	Whole-genome sequencing of mutant and functional validation in *P. domestica*^155–157^	1.8 kb deletion spanning the gene’s 5’ end
Broomy (pillar)	*P. persica*	G2 (*Br*)	Tiller angle control (*PpeTAC1*, *ppa010082; Prupe.2G194000*)	Sequencing‐based mapping strategy, whole-genome sequence analysis, expression analysis, and CG homology with Arabidopsis mutant^156^	Insertion producing a nonsense mutation
Gametophytic self-incompatibility (pistil)	*Various Prunus*	G6 (*S*)	S-Ribonuclease (*S*-*RNAse*)	Allele cloning, genotyping, and self-compatibility screening^160,161^	–
Gametophytic self-incompatibility (pollen)	*Various Prunus*	G6 (*S*)	F-box protein (*SFB*)	Allele cloning, genotyping, and self-compatibility screening^165,166^	–
Gametophytic self-incompatibility (pollen)	*P. armeniaca*	G3 (*M*)	Disulfide bond A-like oxidoreductase (*ParMDO*)	Fine mapping, gene expression analysis, whole-genome resequence analysis^163^	Miniature inverted-repeat transposable element (MITE) insertion
Double flower	*P. persica*	G6 (*Di2*)	EU-AP2 transcription factor, TOE-type (*Prupe.6G242400*)	Fine mapping, whole-genome resequence analysis, and CG functional classification and expression analysis^242^	Deletion of an miRNA target site

*GWAS* genome-wide association study, *QTL* quantitative trait locus, *SNP* single-nucleotide polymorphism, *SSR* simple sequence repeat

^a^In parenthesis, major gene name

^b^Genes starting with *ppa* and *Prupe* correspond to the annotation of the v1.0 and v2.0 versions of the peach genome, respectively; *Pav* are genes from the sweet cherry genome v1.0

The public availability of a peach high-quality DNA sequence in 2010^10^ and later those of other *Prunus* species^11–13^ unlocked the *Prunus* genome, paving the way to build new knowledge for basic and applied purposes. Our objective in this paper is to review the major achievements that have taken place in the genetics of peach and other *Prunus* crops as a consequence of the availability of whole-genome sequences (WGSs), emphasizing the new genes discovered, and in future targets that will allow us to reach a much deeper understanding of the genetics of a group of species that are important sources of high-quality and healthy nutrients and serve as models for tree species.

## The peach genome sequence and other *Prunus* genomes

The plan to sequence the peach genome emerged in the middle of the past decade. Peach was an excellent candidate for shotgun sequencing having a small genome estimated at 265 Mb^14^ and extended genomic and genetic resources, such as linkage maps, EST collections, and BAC libraries^4^. The decision to sequence the peach genome was announced by the Joint Genome Institute in 2007 and soon after an Italian consortium (DRUPOMICS) joined the US efforts to form the International Peach Genome Initiative (IPGI) that included scientific institutions from Chile, Spain, and France as well.

The use of whole-genome shotgun sequencing with Sanger chemistry and a completely homozygous genotype, the “Lovell” double haploid PLov2-2n, were crucial for the success of the initiative. Sequencing concluded in late 2009 with the deposition of 3,729,679 paired-end Sanger sequences corresponding to 8.47× genome coverage. Assembly and annotation of genes and repeated sequences were finished in early 2010 and the peach genome sequence was released, under a Fort Lauderdale agreement on April 1, 2010, being the first freely available Rosaceae genome. The assembly^10^ was arranged in 2720 contigs and 234 scaffolds covering 227.3 Mb. Scaffolds were assigned to chromosomes using an improved version of the *Prunus* reference map^2,15^ to form a chromosome-scale assembly with 218.3 Mb (96%) of sequences in 8 pseudomolecules and 194 unmapped scaffolds (Table 2), providing a sequence accuracy of 99.96% and a completeness of 99%. A combination of de novo identification and structural tools together with similarity searches was used for the annotation of the 84.4 Mb (37.14%) of repetitive sequences. Several homology-based predictors, incorporating transcript assemblies from Rosaceae and *Prunus* ESTs and protein homology with the major sequenced plants, were used to derive 27,852 protein-coding genes and 28,689 protein-coding transcripts. Comparative analyses with other sequenced species and the detection of duplicates within the genome itself highlighted that peach has not undergone recent whole-genome duplication after the ϒ Eudicot hexaploidization^10^.

**Table 2:** Comparison of the assembly and statistics among the *Prunus* genomes so far published

	Peach v2.0	Peach v1.0	*P. mume*	PAV_r1.0 (*P. avium*)	*P. yedoensis*
Sequencing strategy	Sanger–NGS Illumina	Sanger	NGS Illumina	NGS Illumina	NGS Illumina–PacBio
Genome coverage	8.47×–NGS 64×	Sanger 8.47×	180×	130.34×	1265.6× (1192× + 73×)
Scaffold number total	191 (241)^a^	202 (234)	1449	10,148	3185
Contig number total	2525	2730	10,894	–	4292
Scaffold sequence total	227.4 Mb	227.3	237.1 Mb	272.4 Mb	323.8 Mb
Mapped scaffold sequence total	225.7 Mb (99.2%)	218.4 Mb (96%)	198.9 (83.9%)	191.7 Mb (70.4%)	281.6 Mb (87.0%)
Oriented scaffold sequence	223.3 Mb (98.2%)	194.6 Mb (85.6%)	–	–	–
Contig sequence total	224.6 Mb	224.6 Mb	219.9 Mb	–	318.7 Mb
Scaffold N50/L50	10/7.3 Mb	9/8.9 Mb	120/577.8 kb	–/219.6 kb	519/198.9 kb
Scaffold N50/L50 (chromosome-scale assembly)	4/27.4 Mb	4/26.8 Mb	–	–	–
Contig N50/L50	250/255.4 kb	294/214.2 kb	2009/31.8 kb	–/276 bp	706/132.6 kb
Number of scaffolds >50 kb	11	21	–	–	–
% main genome in scaffolds >50 kb	99.4%	99.4	–	–	–
Repetitive sequences	79.38 Mb (35.34%)	84.41 Mb (37.14%)	106.8 Mb (44.9%)	119.4 Mb (43.8%)	150.8 Mb (47.2%)
Protein-coding genes	26,873	27,852	31,390	43,673	41,294
Protein-coding transcripts	47,089	28,689	–	–	45,581
Alternative transcripts	20,216	837	–	–	4287

^a^In brackets, assembled scaffolds prior the build of pseudomolecules

Further analyses of the peach WGS suggested several areas for improvement, including unmapped and randomly oriented scaffolds; correction of putative misassemblies; and better base accuracy, contiguity, and gene prediction. These goals were achieved in the second version of the peach genome (Peach v2.0)^16^ using a combination of high-density linkage maps (4 maps and 3576 markers) and high-throughput DNA and RNA sequencing. The improved chromosome-scale assembly is now 58 scaffolds spanning 225.7 Mb (99.2%) arranged in 8 pseudomolecules, 52 of which are oriented (223.3 Mb; 98.2%). Sixty-four-fold Illumina whole-genome sequencing of the reference genotype was used to correct 859 false single-nucleotide polymorphisms (SNPs) and 1347 indels. Moreover, the assembled next-generation sequencing (NGS) contigs enabled the closing of 212 gaps with a 19.2% improvement in the contig L50. Gene annotation was improved using a large amount of RNA-seq data from various peach tissues and organs, as well as annotated repeats that include low copy repeats and a complete set of Helitron transposons (Table 2).

Two other *Prunus* WGSs (Table 2) were obtained using high-throughput NGS Illumina sequencing: (1) Japanese apricot (*P. mume)*^11^, producing a fragmented assembly of 237 Mb with 31,390 protein-coding genes and 45% (106.8 Mb) of repeated sequences; and (2) cherry (*P. avium*)^12^ with a total length of 272.4Mb, containing 43,349 protein-coding genes and 119.4 Mb (43.8%) of repetitive sequences. Third-generation sequencing methods based on single-molecule sequencing (such as Pacific Biosciences, Oxford Nanopore, and Moleculo) capable of obtaining long reads, up to 50 kb, coupled with short read NGS technologies have provided new inputs to genome sequences. Recently, using a combination of ultra-high coverage (1265.6-fold sequences) Illumina and PacBio long read methodologies, the wild cherry *P. yedoensis* genome was sequenced^13^, producing an assembly of 323.8 Mb with 41,294 protein-coding genes (Table 2). The sequence of “Texas” almond (P. Arús, unpublished results) is already publicly available at the Genome Database for Rosaceae (GDR).

Other de novo assemblies of *Prunus* genomes are underway, such as that of another sweet cherry cultivar, “Regina” (E. Dirlewanger, unpublished results), while those for other *Prunus* crops, plum, apricot, and other wild crop relatives are expected to be released in the next several years. These new sequences provide an opportunity to unravel genetic diversity—still present in *Prunus* wild species—that had been lost in these crops during thousands of years of selection and breeding. This genomic information could be exploited by introgressing new desirable alleles into the cultivated species via classical or marker-assisted breeding (MAB) methods. It could also be the starting point for applying gene editing and cisgenesis to *Prunus* crops in the efforts to create new targeted variability in genes controlling relevant agronomic traits, such as biotic and abiotic stress resistance, for which the cultivated species, peach in particular, are notably lacking.

Soon after the release of the peach genome sequence, the International Peach SNP consortium developed the first peach 9K SNP array v1^17^. Furthermore, the extensive *Prunus* synteny^2^ made the peach genome a valuable reference to identify SNPs in related crops^10,18^ enabling the construction of a cherry 6K SNP array^19^. Recently the peach and cherry arrays have been updated with 9000 additional SNPs covering previous gaps (I. Verde, unpublished results). In addition to SNP arrays, resequencing data from sets of individuals has enabled unprecedented in-depth analyses of variability and linkage disequilibrium (LD)-based analysis that resulted in the first genome-wide association studies (GWASs). Such applications of the *Prunus* WGSs are reviewed below.

## Whole-genome analysis of *Prunus* diversity

### *Prunus* variability, domestication, and crop evolution

The most comprehensive *P. persica* diversity analysis was performed with the 9K peach SNP array^17^ and a collection of 1580 accessions (including peaches and few closely related *Prunus* species)^20^. SNP data identified some unknown somatic mutants and confirmed others that were already reported. In agreement with the known breeding history of peach and previous results with simple sequence repeats (SSRs)^21,22^, the unique accessions showed a relatively high kinship coefficient corresponding to an average relationship of grandparent–grandchild or half-sibs and were divided into three main subpopulations: two based on geographic distribution (oriental and occidental accessions) and the third, a separation within occidental materials as traditional landraces or those derived from breeding programs. The cherry 6K SNP array^19^ was used to characterize 210 cherry accessions. As for peach, variability in sweet cherry was differentially distributed between cultivars and landraces and, in the latter, among geographic locations^23^.

Whole-genome resequencing of germplasm collections has also been used for variability analysis, particularly in peach, including cultivars with variability underrepresented in the cultivar panel used for the development of the 9K SNP array (such as those from Asian germplasm collections) and *Prunus* wild species. In addition, such high level of genome definition has provided relevant knowledge on domestication and crop evolution processes. Most *Prunus* cultivated species originated in central Asia, although their recent evolution seems to have followed substantially different patterns. For example, peach and almond, two of the genetically closest relatives, seem to have arisen from a common ancestor in central Asia by the time of the uplift of the Central Asian Massif around 8 MYA^24,25^, and then followed completely different paths, almond in the dry environments of the central and western Asian steppes and peach in the humid, subtropical climate of south-western China^24^. Important trait differences accumulated such as dry vs. fleshy fruit and outcrossing vs. selfing, with many derived biological and populational consequences^25^. Recent comparison of genome-wide diversity patterns in almond and peach has identified regions with signatures of selection that occurred during domestication that interestingly often overlap in both species despite having occurred independently in each around 5000 years ago^26^. Likewise, during *P. persica* domestication, which probably took place through a single domestication event from wild peaches^27^, some genes were subjected to artificial selection as deduced from comparisons of the regions showing selective sweeps among domesticated peaches (including landraces, edible and ornamental cultivars) and their wild relatives^27–29^. Such regions contain genes that may have contributed to peach domestication and cultivar differentiation and warrant further research. Although interspecific hybridization events, such as those accompanying creation of some *Prunus* crops like Japanese plum^30^, might be associated with increased mutation rates, low evolutionarily rates reported for perennial plants, peach in particular^31^, may explain the close relationships among *Prunus* species.

Although SSRs and SNPs are and will remain the principal genotyping tools in *Prunus* species lacking a reference genome, the continuous cost reduction of sequencing techniques forecasts the routine use of resequencing data in future *Prunus* variability studies. Currently, increased sequencing efforts are feeding the databases and providing important sequence resources for diversity and domestication analyses. In a near future, we may expect an increasing number of studies focused on the identification of particular genomic regions, such as those showing differential patterns of diversity as a consequence of domestication or breeding, gene families, or DNA features such as transposable elements (TEs). Such studies may answer fundamental questions concerning when, where, and how domestication events took place in the development of certain *Prunus* species. However, although promising, relying on available sequence data has a major drawback since most has been done with resequencing techniques that yield short reads and with low depth. Their alignment against the “Lovell” reference genome may fail to map genetic regions that have been lost or duplicated during the domestication process. The recent development of new long-range sequencing technologies should resolve this problem.

Whole-genome diversity has been exploited in GWAS using both SNPs and whole-genome resequencing data with a minor or major depth coverage. Understanding the extension and decay of LD in the species of interest, and in particular in the panel of genotypes used for association analysis, is essential when considering GWAS. WGS data has identified fast LD decay in apricot, spanning <100 bp^18^. Similarly, LD decays fast in ornamental *P. mume* accessions (*r*^2^ ≤ 0.2 at 50 kb to few hundreds of base-pairs depending on the population)^32^ and a little more moderately in cherry (*r*^2^ ≤ 0.2 at 100 kb)^23^. In contrast, large LD extensions were observed in peach (*r*^2^ ≤ 0.2 between 0.8 and 1.4 Mb depending on the population^20^). These findings have practical implications: while a large number of SNPs will be required to find some associated with the allele of interest in apricot, lower SNP density should be sufficient in peach. However, as a trade-off, associated SNPs in the former will be most likely closer to the gene than in peach. As a proof of concept, GWAS for several qualitative traits was performed in peach^20^, identifying haplotypes linked to the loci or genes already known to be responsible for the trait. GWAS using resequence data from 129 peach accessions^28^ identified association signals at various loci for a set of 12 agronomic characters and proposed candidate genes for two of them. Recently, the stony hard flesh (*Sh*) locus was mapped in a panel of peach genotypes using the 9K SNP array^33^. A highly associated SNP was found that, due to the extended LD of peach at this chromosomal region consequence of its location close to the centromere, was organized in a haplotype about 1.9-Mb long. In this case, higher SNP density would not have translated to higher precision.

Up to now, GWAS has mainly been performed for agronomic and fruit traits evaluated in a single location, usually with small-scale phenotyping techniques. In the near future, we may expect to see GWAS of genetic traits related to crop adaptation, probably involved in epigenetic variability and genome instability, as already evident in some large-scale *Arabidopsis* studies^34,35^. In addition, thanks to the current inclusion of new technologies in the life sciences field, we may expect that new high-throughput phenotyping methods such as those based on three-dimensional imaging^36,37^ or large-size plant mobile screening platforms^38^ will soon provide enormous amounts of data as substrate for future GWAS.

Disadvantages of the multi-year intergenerational period of *Prunus* species, the seasonality of crop production, and large plant sizes are balanced by the ability to clonally propagate and maintain heterozygous genotypes for many years. This enables evaluation of the same genotype under different climatic and management conditions. Future GWAS strategies should consider the analysis of existing genotype collections replicated in different locations to incorporate environmental effects in the analysis.

### Haploblocking *Prunus* genomes

**Fig. 1:**
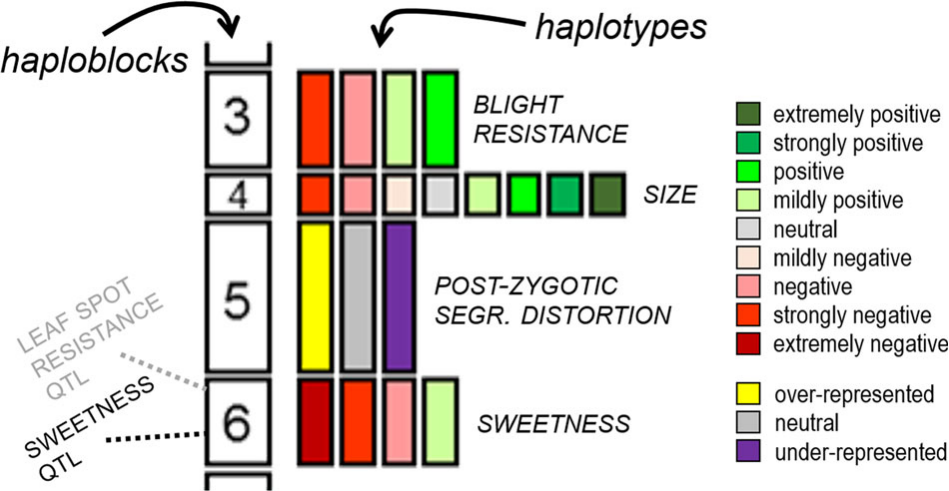
Dividing a crop’s genome into qualitative, cognitively manageable segments by haploblocking adjacent sets of loci can be done in several ways, such as the pedigree-based approach, in which loci within haploblocks have not recombined throughout the pedigree of known progenitors of cultivars. Haplotypes are the variants of haploblocks—sets of co-inherited alleles. To each haplotype can be assigned trait influences, ancestry, and other genetic features. If a breeding parent does not have coupling-phase linkage for desirable alleles within a haploblock containing multiple quantitative trait loci, such tight linkage might be targeted for recombination in the next generation

The use of high-density SNP and resequencing data allows high-resolution genome analysis such as “haploblocking”, or haplotype blocking, a means of dividing the genome of *Prunus* crop individuals into informative genetic segments. Each segment (called a haploblock) has variants (called haplotypes) within and across individuals. Alleles of the loci within each haplotype tend to be co-inherited owing to their close linkage. Functional utility can be assigned to haplotypes, such as trait effects and ancestral origin. The managers of haploblocked genomes are geneticists and breeders, and so the patterns of co-inheritance described by haploblocking are designed to capture features of value to such users (Fig. 1). Haploblocking provides a fascinating overview of the genetics of any individual examined.

At the broadest level, whole chromosomes are haploblocks, described by their genetic length in centiMorgans (cM), but a finer level of compartmentalization is possible and desirable. In practice, haploblocking divides up chromosomes, establishing systematic criteria for helpfully delimiting each haplotyped region. The series of haploblocks along each chromosome act as an easily manageable number of linked multi-allelic loci, carrying all the information of the underlying genotypic data^39^. A requirement for haploblocking is phased, high-resolution genotypic data, which is currently obtained by genotyping individuals with high-density SNP assays and correct phasing for each individual that is achieved, for example, via inheritance analyses using the software FlexQTL™^40^.

There are several ways that *Prunus* crop haploblocks have been or are being delimited. The pedigree-based approach identifies positions of historical recombination in the pedigreed germplasm and uses these positions to delimit haploblocks. In U.S. breeding germplasm of sweet cherry^41^ and peach (C. Da Silva Linge, pers. comm.), recombination positions used to define haploblock boundaries were all of those detected in the known progenitor generations leading to cultivars of the germplasm set. Recombination events were positioned with VisualFlexQTL^40^, and finally haplotypes were assigned to each haploblock using PediHaplotyper^39^. As germplasm managers using pedigree-based haploblock information examine haplotypes through ancestral generations, they do not have to track complicating recombinations within haplotypes—there is 100% LD within haploblocks. Note that this coupling-phase linkage within a haploblock can be broken into subsequent generations, with a probability determined by the genetic distance between loci. Other ways to establish haploblocks include using regular, arbitrary lengths for each haploblock, such as 1 or 5 cM; the diversity-based approach, whereby the observed degree of LD along each chromosome at increasing levels of genetic diversity among sets of individuals, from families to across species and even across genera, are used to define segments that have rarely recombined (A. Lawton Rauh, pers. comm.); and the aggregate-marker approach, which combines into haploblocks sets of markers positioned within very short physical distances, enabled by purposeful SNP clustering around focal points as done for design of the 20K apple SNP array^42^ and the upcoming 9 + 9K peach and 6 + 9K cherry SNP arrays. WGSs are a critical foundation of haploblocking, as they determine the genomic positioning of SNP markers to ensure that each chromosome is covered with genetic polymorphism data. Although haploblocks are described in cM, physical anchoring to the WGS ensures ready connection to associated DNA sequences, sequence motifs, and annotated genes. Research community consensus on the positions and labels of a standardized set of haploblocks for each crop should provide a framework for comparative genetics that is easier to use than a reference genetic map of common markers. A proposed basis for such a haploblock framework is the pedigree-based haploblocks derived from the set of 50–100 ancestors that are common to most cultivars of a crop.

Haplotypic variation across haploblocks is powerful genetics information. Haplotypes are genotypically distinguished by differences in their set of alleles (ultimately DNA sequence variation), such as ABBAABB vs. ABABBBB vs. BBBBBBB. These haplotypes can also be functionally distinguished. Where major genes or QTLs for traits of interest lie within (or span) a haploblock, the associated effects of each haplotype on such traits can be calculated and the information attached. Each haplotype can also be traced through the pedigree from the earliest known ancestor that contributed the haplotype through to all descendants carrying that identical-by-descent haplotype. Visualizing the “haplotype mosaics” of this shared ancestry^43^ can provide a valuable experience of elite genomes (section “Applications of genome information in *Prunus* improvement: MAB, MAI, genomic selection, and visualization of the genome-wide genetics of elite individuals”). Identity-by-state among germplasm of one or more haplotypes extending over large genetic distances can be used to infer recent shared common ancestry. Other features relevant to breeding and biodiversity management can also be assigned, such as haplotypes that are overrepresented or under-represented in offspring that thereby contribute to segregation distortions, excessive heterozygosity, and excessive homozygosity. Examining the ancestral origins and germplasm distribution of trait locus alleles or segregation-distorting alleles is a relatively simple task using haploblocks.

Haploblocking is a newly available approach to make sense of the vast quantity of genome-wide genotypic data that can now be readily obtained on germplasm individuals. Critically, it involves the consideration of each individual in genetic comparisons, examining the haplotypes that each carries, rather than simply using scaled-up quantitative analyses. New software in data curation and graphical genotyping is needed to facilitate a multitude of practical applications that beckon in breeding and biodiversity management.

## Genetics of agronomic characters

During the recent decades, many efforts have been directed to identify loci and the underlying genes that explain the phenotypic variability of a number of agronomic traits. Thanks to the availability of reference genomes, once the chromosomal position for a major gene or QTL is established, it is possible to easily discover and develop additional markers that enable reducing the size of the target region, where a small set of genes can be identified. This approach, supported by many RNA-seq datasets available for most *Prunus* species^10^ and epigenetic analysis, has led to the discovery of several causal genes for certain traits and of strong candidates for others in peach and other *Prunus* (Table 1) that are reviewed below.

### Phenology (blooming and maturity dates) and climate change adaptation

In *Prunus* species, as for woody perennial plants in temperate and boreal zones, survival and production depend on precise timing of growth and rest periods in synchrony with seasonal changes in temperature^44^. Flower bud differentiation takes place at the end of the summer and buds continue their development until mid-autumn, when they enter into the dormant stage^45^. The first step of dormancy is growth cessation and meristem acclimatization to cold. Dormancy can be separated into two main phases: (1) endodormancy, when meristems are unable to initiate growth under favorable conditions, followed by (2) ecodormancy, when meristems can resume growth if temperatures are optimal^46^. During endodormancy, a certain amount of cold temperatures, defined as chilling requirement (CR), must accumulate prior to the bud being released from endodormancy. Subsequently, the bud will respond to heat, leading to flowering when heat requirements (HR) are satisfied^46^. Consequently, flowering date is determined by CR and HR, and fruit/nut maturity timing will be associated with flowering date and to the length of the fruit development period.

In the context of global climate change, flowering phenology of deciduous tree species is crucial as it may affect commercial productivity. In fruit tree orchards, flowering phenology has an indirect influence on spring frost damage, pollination, maturity, and fruit production. Climate warming delays the endodormancy release while it accelerates bud growth. Therefore, depending on the balance between these two antagonistic effects, budbreak can be either advanced or delayed and can even be compromised owing to insufficient cold temperature during winter. Additionally, warm temperatures in spring and summer are responsible for an advance of the maturity date. Consequently, it is crucial to anticipate the impact of climate warming on phenological processes with the aim to establish prospective strategies to create new cultivars that are well adapted to future climate. Hence, numerous studies are aimed at deciphering the molecular mechanisms underlying the regulation of bud dormancy and flowering and maturity dates.

Many *Prunus* major genes and QTLs have been described for phenology-related traits such as CR, HR, and flowering and maturity dates. For bloom date, major QTLs were detected on linkage group 6 (G6) for peach and on G4 for apricot and sweet cherry, whereas for maturity date, a major QTL was detected on G4 for all three crops^47^. Fine mapping of the major locus controlling the maturity date in peach identified a precise QTL interval of 220 kb and enabled the identification of a NAC transcription factor as the candidate gene^48^ (Table 1) confirming results previously described^47^. With a multi-progeny mapping strategy, 18 peach progenies in total, additional QTLs were detected for flowering date (G4, G6), maturity date (G2, G4, G6), and for fruit development period (G4, G6)^49^. In sweet cherry, one stable QTL for CR and bloom date was detected in the same region on G4^50^. Candidate genes underlying this QTL were investigated using the peach genome sequence and key genes were identified for these two traits^51^. Most of the genes were those involved in chromatin remodeling (*ARP4*, *EMF2*, *PIE1*) and in gibberellin homeostasis (*KS* and *GA2ox*). Thanks to the high synteny existing among *Prunus* species^2^, it was possible to use the peach and the sweet cherry genomes to compare homologous regions such as the QTL on G4 controlling CR in peach, sweet cherry, and Japanese apricot^52^.

Expression of the dormancy-associated MADS-box (*DAM*) genes has been extensively analyzed and their key role in the endodormancy establishment and maintenance was demonstrated. *DAM5* and *DAM6* genes are upregulated during growth cessation and downregulated by cold exposure during winter in peach^53^, Chinese cherry^54^, sweet cherry^55^, and Japanese apricot^52,56,57^. In peach, histone modifications of *DAM* gene expression were reported^58^. In sweet cherry, DNA methylations and small interfering RNAs involved in the silencing of *PavMADS1* during cold accumulation and dormancy release were analyzed^59^, and research on the epigenetic regulation of the *DAMs*, through chromatin immunoprecipitated-sequencing (ChIP-seq) analyses, is in progress^55^.

New methods that resolve phase-switches and reconstruct contig-length phase blocks^60^ make it possible to identify the haplotypes for each individual, which are especially useful for highly heterozygous genotypes. This ability is important for phenological traits, which are often complex, highly polymorphic, and may involve epigenetic regulation. Genomic selection can be a useful breeding strategy for the phenological traits and promising results were obtained for apricot^61^ and sweet cherry^62^ for flowering and maturity dates. In addition to these tools, spontaneous mutants are highly valued for identifying genes responsible for phenotypic variation. Many spontaneous (epi-)mutations were found in *Prunus* species^63^ and several of them were associated with flowering date. In sweet cherry, the very early-flowering cultivar “Cristobalina” was issued from a spontaneous mutation of “Temprana de Sot”^64^. In apricot, early-flowering “Rojo Pasión Precoz” was derived from the recently released “Rojo Pasión” apricot cultivar^65^. To date, no clues about the determinism of these extreme phenotypes are available. Using reference WGSs and resequencing data of these mutants or of cultivars with contrasting phenotypes, it should be possible to identify the causal genes.

### Fruit-related characters

#### Fruit size, shape, and color

Fruit appearance highly influences consumer’s choice. Therefore, large fruits, with a certain fruit color and shape are breeder’s priorities. Fruit size is one of the most important phenotypic traits that distinguishes modern *Prunus* cultivars from their small-fruited wild ancestors. For example, in sweet cherry, wild (syn. mazzard), landrace, and modern bred cultivars typically have fruit sizes of ~2, 6, and 12 g, respectively^66^. Discovering major loci underlying genes that determine fruit size has been a high priority due to the importance of fruit size to growers’ profitability and the need for breeders to select against small-fruited alleles when introgressing traits from wild *Prunus* species.

Fruit size is a classic quantitative trait controlled by many loci, highly influenced by the environment, with >50 QTLs identified to date. Many fruit size studies have involved crossing large-fruited modern cultivars with *Prunus* wild relatives, which in peach consisted of crosses with *P. davidiana*, an ornamental small-fruited *P. persica* and almond *P. dulcis*^49,67–69^. A comparison of the nucleotide diversity in genome sequences of these wild species (*P. kansuensis* and *P. davidiana*) with that of cultivated peach identified regions associated with selective sweeps related to domestication and breeding^10^. In sweet cherry, a fruit size QTL region on chromosome 2 was shown to be under positive selection^41^. In certain cases, QTLs co-locate across species such as the G7 QTL discovered in both peach^68^ and Japanese plum^70^. In other cases, the co-location of fruit size genes with fruit firmness, such as the QTL on G5 in cherry^71^, raises the question of whether this is a pleiotropic locus. However, it will take the discovery of the underlying genes to know whether there are one or more genes responsible for these QTLs.

To date, members of two gene families have been associated with the fruit size increase that accompanied domestication. The first study involved the *FW2.2* cell number regulator (CNR) gene family originally identified in tomato^72^. A total of 23 *FW2.2/CNR* family members were identified in the peach genome, two of these *CNRs* co-located with fruit size QTLs on G2 and G6 in a domesticated sweet cherry × mazzard cross^73^ (Table 1). The gene family member on G2, named *PavCNR12*, was hypothesized to contribute to an increase in fruit size by increasing mesocarp cell number. The gene family member on G6, *PpCNR20*, is also a candidate gene for a fruit weight QTL identified in peach^74^. Another gene implicated in the control of fruit size in sweet cherry is a member of the Cytochrome P450 (CYP) subfamily, termed *CYP78A* that has been shown to be involved in plant organ growth and development including tomato fruit^75^. The transcript level for *PaCYP78A9* was significantly higher in a cherry landrace compared to a mazzard cherry^76^. In addition, silencing of *PaCYP78A9* during sweet cherry fruit development caused a reduction in fruit size due to a reduction in both mesocarp cell number and size. To date, this gene has not been shown to be associated with a fruit size QTL.

**Fig. 2:**
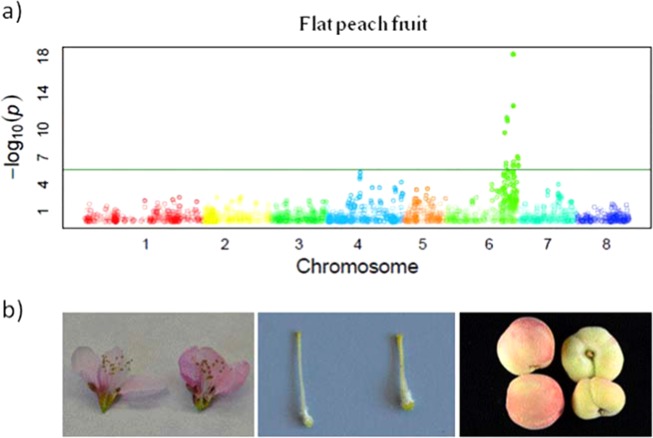
Flat fruit shape in peach. **a** Manhattan plot from Micheletti et al.^23^ data. Chromosomes are marked with different colors on the horizontal axis. The horizontal green line represents the significance threshold for the association. **b** Images of flowers, pistils, and fruits of a round (left) and a flat (right) fruit cultivar where it can be seen that the flat vs. round character is determined early in flower formation

*Prunus* fruits show large intraspecific variability for fruit and stone shape, which can be used for germplasm characterization. Similar to fruit size, cell number may determine fruit shape. This is the case of the flat shape of peach fruits, which is primarily determined by the regulation of cell production in the vertical direction during early fruit development^77^. The flat shape locus *S/s* has been mapped on G6, where *S* is a partly dominant allele; *ss* genotypes produce round fruits, *Ss* genotypes produce flat fruits and fruits from *SS* trees abort a few weeks after fruit set^78^. The participation of a second gene producing fruit abortion has been hypothesized, although pistils of *SS* flowers show already a differential phenotype compared to *Ss* and *ss* types (Fig. 2). Association analysis of SNPs and indels in the *S* locus suggests the gene *Prupe.6G281100*, a leucine-rich receptor-like kinase (LRR-RLK), as a candidate for the fruit shape (Table 1). The best protein hit of this gene occurred with *AtRLP12*, which in Arabidopsis functionally complements *CLAVATA2*, a key regulator that controls the stem cell population size^79^. The flat-associated allele of *Prupe.6G281100* is not expressed, resembling a dosage mechanism. However, polymorphisms in this gene do not completely explain the trait in all germplasm, suggesting the participation of additional alleles or genes^77,79^. Apart from the peach flat shape, investigation of genes determining diverse shapes (i.e., oblate or elliptic) is still lacking.

The inheritance of white vs. yellow flesh in peach, one of the first Mendelian traits described in *Prunus* (*Y*/*y*), maps to G1^80^ with white flesh dominant over yellow flesh. The pigments abundant in the yellow peaches are carotenoids, for which the biosynthetic pathway is well known. Subsequently, the gene underlying the *Y* locus was shown to be a carotenoid cleavage dioxygenase 4 (*PpCCD4*) that is considered responsible for carotenoid degradation in white-fleshed peaches^81^. The dominant functional allele was found to confer white flesh while yellow peaches have loss-of-function mutations of this gene^82^ (Table 1).

Anthocyanin pigments in *Prunus* are responsible for the red fruit skin and flesh color including the red “blush” on peach fruit. Their biosynthetic pathway is well studied in many crops including apple where a R2R3 MYB anthocyanin transcription factor, *MdMYB10*, controls red fruit flesh color^83,84^. Likewise, in *Prunus*, there is strong evidence that this same anthocyanin transcription factor, *MYB10*, on G3 is the major gene responsible for the red color differences (Table 1). In sweet cherry, mahogany fruit (skin and flesh) color is dominant to yellow fruit color and the major QTL on G3 maps to an interval containing *PavMYB10*^85^. This same G3 interval was shown to have major genes/QTLs controlling anther color in almond × peach progenies^69^, red blush on the skin in peach^86,87^, and skin color in Japanese plum^70^. However, other QTLs controlling the percentage of red skin color in peach were identified on G4, G5, and G6^49^. Some of these QTLs have been shown to exhibit epistatic interactions^85^, and this has been extended to interactions among the involved genes. For example, the red-flesh peach type, called “blood type,” was found to be controlled by multiple loci with a candidate gene identified on chromosome 5 that regulates the transcription of *PpMYB10*^88^ (Table 1).

Owing to an excellent understanding of pigment biosynthesis pathways and the high heritability for color, the molecular characterization of fruit color is the most advanced. Yet, knowledge on the molecular basis of the more subtle differences in color will require the identification of the full set of genes and an exploration of epistatic interactions. For example, a transcriptomic analysis of fruit ripening in plum revealed the co-expression of the MYB transcription factor with another important anthocyanin transcription factor, a basic helix-loop-helix, bHLH^89^.

The second trait for which the identification of likely candidate genes is most advanced is mesocarp cell number where the comparisons were made among representatives of wild and domesticated germplasm. Characterizing the alleles for candidate genes is an important next step made possible by the increasing amount of sequence data from a broader range of plant materials. This enables proof-of-function experiments such as those described for the sweet cherry fruit size gene *PaCYP78A9*^76^. These types of experiments are also critical for addressing hypotheses of pleiotropy that have been suggested for a few co-locating QTLs. In addition to narrowing QTL regions to reduce the number of candidate genes, fine mapping studies are needed to dissect the trait-rich QTL regions. Obvious first targets are the trait-rich clusters on chromosome 2 in sweet cherry^41^ and chromosome 4 in multiple *Prunus* species^70,90,91^ (A. Iezzoni, unpublished results).

#### Fruit flavor and flesh consistency

Apart from the external appearance, sensorial attributes (like flavor and flesh consistency) are highly relevant for fruit quality. *Prunus* fruit flavor is dependent on the concentrations and balance of sugars and acids. Elucidating the genetic control of these metabolites is difficult due to the metabolic complexity, changes in individual sugar and acid levels during fruit maturation, and the environment. Numerous QTLs have been identified on all eight *Prunus* chromosomes for traits and metabolites associated with sweetness (e.g., soluble solid content (SSC), individual sugars: fructose, glucose, sucrose and sorbitol), and acidity (e.g., pH, titratable acidity, and malic acid). In peach, one QTL mapped to the top of G5 behaves as a major locus, *D*/*d*, determining low acid (subacid) content^92–94^. In general, fruit can be placed into two groups, normal acid and low acid, with low acidity determined by the dominant allele. The physical map location of the *D* locus has been narrowed to a 100-kb region^93^ and a candidate gene coding for an auxin afflux carrier family protein (*ppa006339m; Prupe.5G004300*) was proposed based on GWAS^28^ (Table 1). For the components of sweetness, many QTLs have been identified most with minor effects. For example, six QTLs for SSC were found in a collection of 18 populations of peach^49^, and numerous QTLs for sugar metabolism during peach fruit development along with co-localized candidate genes have been reported^95^. Yet, the determination of the underlying genes and their metabolic functions is in its infancy. Global mRNA and protein profiling results can provide support for candidate genes and identify key metabolic switches and co-expression gene networks^96^ controlling the metabolism and accumulation of compounds contributing to fruit flavor. This approach may be particularly useful for sweetness, as the vast majority of the QTLs identified have minor effects. It was successfully used to characterize differences in sugar metabolism pathways between two Japanese plum cultivars, the climacteric “Santa Rosa” and its non-climacteric bud sport mutant^97^, resulting in the identification of several highly connected sugar metabolism-associated genes that could be acting as metabolic hubs. These highly connected genes could be targets for future study.

Fruit flesh texture is one of the key traits in most peach breeding programs. On one side, it determines two different fruit typologies: melting flesh (MF) for fresh consumption, and non-melting (NMF), mainly for canning. On the other side, flesh texture also determines postharvest behavior, with other fruit texture types as slow melting (SMF) and stony hard (SH), both associated with longer shelf life. The *M* and *F* genes determining fruit texture (MF/NMF) and flesh adhesion to the stone (clingstone vs. freestone), respectively, were identified very early as endopolygalacturonase (endoPG) genes^5,6^ (Table 1). They map to a single locus on G4, where two endoPG genes, *Prupe.4G261900* and *Prupe.4G262200*, are involved in their inheritance. The different haplotypes of these two loci and the phenotypes that they determine have been elucidated^6,98^. Using this information, different molecular markers sets have been developed^99^ (I. Eduardo unpublished results) to genotype this locus and to identify the alleles that determine the different flesh consistency types, including freestone melting flesh, clingstone melting flesh, clingstone non-melting flesh, and clingstone non-softening flesh.

The SMF phenotype is defined by a slower process of postharvest fruit-softening than the MF types, with “Big Top” being the reference cultivar for the trait^100^. Using high-density genetic maps of two populations with “Big Top” as one of the parents, two QTLs involved in the trait were identified in G4 and G5, both affecting fruit-softening rate and maturity date^101^. The QTL on G4 co-localizes with a gene determining the slow ripening trait, a recessive mutation that determines the production of fruit that never ripe^102^, and with the major QTL determining the maturity date described in section “Phenology (blooming and maturity dates) and climate change adaptation.” The *Prupe.4G186800* gene, coding for a NAC transcription factor, was proposed as a strong candidate for the causal gene of these traits^48^. The second QTL, mapping on G5, was detected only in “Big Top” and also co-locates with a QTL for maturity date, overlapping with a region where other NAC transcription factors are located.

Another trait related with peach flesh texture is SH, which presents a crispy flesh when fully ripe and is known to be controlled by a single gene (*Hd*/*hd*). Based on digital gene expression analysis, a YUCCA flavin mono-oxygenase gene (*PpYUC11-like*, *ppa008176m*) located on G6 was proposed as a candidate gene for *Hd* (Table 1), and an SSR in the intron of this gene was identified as a possible diagnostic marker^103^. Later on, this gene and this marker were confirmed using GWAS^33^ and the insertion of a transposon-like sequence was detected upstream of the PpYUC11 gene in the *hd* allele, suggesting its causal effect for the SH phenotype^104^.

To have a complete picture of the genetic control of peach texture, it is necessary to: (1) identify the alleles of the genes involved in this trait to better understand how these genes interact with each other and with other important genes, such as the ones determining maturity date; and (2) understand how these gene interaction networks are influenced by the environment. Achieving all these goals relies on improved higher-throughput phenotyping methods that increase the power to discriminate the different texture types and the number of samples analyzed. This will require appropriate populations or germplasm collections to be evaluated and the development, where necessary, of plant resources specifically bred to facilitate phenotypic analysis of this important character.

#### Fruit allergens

Fruit allergy is becoming a significant health issue. There have been frequent reports of peach fruit allergy globally, especially in China and the Mediterranean countries where peach consumption is high and *Artemisia* pollen allergy is prevalent^105^. Peach-related allergic symptoms include the oral allergy syndrome, urticaria, gastrointestinal symptoms, and anaphylaxis. Sensitization to peach lipid transfer protein allergens also results in broad cross-reactivity to other fruits and plant foods^106^. Five allergens in peach have been identified: Pru p 1 (pathogenesis-related protein, PR-10), Pru p 2 (thaumatin-like protein, PR-5), Pru p 3 (nonspecific lipid transfer protein, PR-14), Pru p 4 (profilin) and Pru p 7 (peamaclein, Gibberellin-regulated protein)^107,108^. With the availability of the peach WGS, all putative peach allergen genes have been located precisely^107^: a cluster of six *Pru p 1* genes, *Pru p 2.04*, *Pru p 4.01*, and *Pru p 7*, are located on peach chromosome 1; *Pru p 2.01* on chromosome 3; three members of *Pru p 3* in a cluster on chromosome 6; *Pru p 4.02* and *Pru p 2.02* on chromosome 7; and *Pru p 2.03* on chromosome 8^109^. Gene expression studies of these genes in fruit, leaf, and anther tissues showed differential patterns and fruit specific genes that have been further investigated under different light-shading treatments^109,110^, demonstrating their variable expression in response to light.

Pru p 1 allergen is cross reactive to birch pollen allergen Bet v 1 in northern of China and Europe, its sensitization resulting in mild oral allergy symptoms. Two isoforms (Pru p 1.01, Pru p 1.02) are expressed in peach fruit and have similar IgE-binding properties^111^. The Pru p 3 allergen can cause a severe food allergic reaction, while different peach fruit types (peach, nectarine) have a variable content of this allergen in peel and pulp^110^. There is no genetic diversity of the *Pru p 3.01* protein-coding sequence in *P. persica*. Based on the peach genome sequence, further cloning and sequencing of the upstream region in peach germplasm identified three different allele sequences, with variable frequencies in different subpopulations^110^. These differences may link to the variation of Pru p 3 content in different cultivars.

There is a need to select hypoallergenic peach cultivars that can be consumed by mild peach allergy patients. Currently about 100 core peach accessions are being evaluated for their Pru p 3 content by a newly developed sensitive method of monoclonal antibody-based enzyme-linked immunosorbent assay^112^. Preliminary results showed multiple genetic and environmental factors influencing the content. In general, nectarine and red flesh peaches have lower Pru p 3 content. Quantification of Pru p 1 content is currently in process. Peach fruit allergens accumulate more during fruit development, especially at the ripening stage. Most of these allergenic proteins belong to pathogenesis-related protein families and have biological functions, such as broad disease resistance. They may also be related to fruit softening and fruit quality traits. Combined omics’ tools used in range-wide peach germplasm samples will provide new qualitative and quantitative insights on these allergens and their impact on other agronomic traits.

### Disease resistance

Host natural resistance is essential for cost-effective and environmentally safe strategies to address the challenges of day-by-day biotic stress. Whereas, initially, stone fruit tree cultivation relied on seed propagation, it is now done by grafting. While vegetative propagation in perennials has the advantage of growing extensively desirable genotypes, cultivars remain unchanged for centuries while pathogens rapidly adapt to them, causing devastating diseases that are increasingly more difficult to circumvent with chemicals and/or eradication measures. Additionally, moving toward environment-friendly and healthy production systems is strongly desired by consumers, demanding transition to a chemical pesticide-free agriculture with limited impact on the environment.

In this context, the use of resistant cultivars is the foremost breeding strategy for crop protection. *Prunus* crop species are susceptible to over 70 pests and diseases, but resistant varieties are not readily available and only a few pathosystems are the subject of research programs. Based on the high-quality peach reference genome and tools developed from it, linkage maps were obtained from F_1_ and F_2_ populations segregating for resistance to brown rot^113,114^, green peach aphid^115–118^, root-knot nematodes^119–122^, fungal gummosis^123^, bacterial spot^124^, powdery mildew^69,118,125^, X-disease^126^, and sharka^18,127–130^. Whereas currently none of the genes and QTLs identified are functionally validated, except for the nematode resistance *Ma* locus^131^ (Table 1), these data can be used to implement MAB for resistance as demonstrated for bacterial spot disease and green peach aphid in peach^117,132^ and sharka in apricot^128,133,134^. A parallel approach to these structurally based genomic analyses is the functionally based identification of differentially expressed genes, in a context of pathogen infection. This strategy relies on the alignment of the RNA-seq reads to the peach reference genome and on its annotation and gene ontology. Only one of those studies based on the transcriptome analysis generated a peach candidate gene that was confirmed in a bacteria/tobacco heterologous system^135^. In spite of this difficulty in functionally validating candidate genes associated with resistance traits, one promising strategy is to search, in the peach reference genome, for orthologous genes that were proven to control resistance/susceptibility to the same pathogen in other hosts^136^. However, this translational research strategy, leveraging knowledge from model plants to *Prunus* crop species, is limited by (1) the significant challenge of genetically transforming stone fruit trees and (2) the acceptance of genetically modified fruits by consumers. Meanwhile, benefiting from whole-genome resequencing in several peach cultivars and other *Prunus* species^119,137–140^, our knowledge on resistance, pathogenesis-related, and defense elicitor gene diversity and evolution in *Prunus* species is increasing.

Breeding for resistance to pests and pathogens in stone fruit crops encounters the usual problems associated with breeding perennial plants. This process is significantly hampered by the complexities inherent to tree testing and evaluation, our lack of knowledge of the pest/pathogen biological interaction, and the diversity and genetic determinism of the target resistance traits that is often quantitative. On top of that, screening for pathogen/pest resistance in stone fruit tree germplasm collections is a very time-consuming and effort intensive task. This likely explains the low number of pests and pathosystems under study, in the past decade. Therefore, there is a great need for academic and practical attention to this area and the development of transnational, high-throughput phenotyping platforms.

The incorporation of resistance genes in current stone fruit breeding programs from related wild species is a promising option. However, it requires significant efforts to identify the valuable germplasm that is unfortunately scarce in the stone fruit crop species. Indeed, excluding resistance mechanisms described in wild *P. kansuensis*, *P. davidiana*, and *P. armeniaca* species^115,119,141^, there is very little information about resistance sources in non-crop *Prunus* relatives. This calls for a more systematic survey of the extent *Prunus* wild relatives around the world, as well as their phenotypic characterization for various resistance traits. These materials could be included in the above-mentioned platforms for high-throughput phenotyping. Once new sources of resistance are identified in wild relatives, the next step is the introgression of the genetic factors linked to resistance into elite *Prunus* cultivars through interspecific crosses. Yet, there are inherent difficulties in doing so that can be minimized by using a marker-assisted introgression (MAI) approach recently proposed and described in section “Applications of genome information in *Prunus* improvement: MAB, MAI, genomic selection, and visualization of the genome-wide genetics of elite individuals” of this paper^142^. Genetic analysis of domesticated and non-domesticated stone fruit genomes may identify the presence–absence variations contributing to trait variation. Toward this goal, a groundbreaking initiative would be the construction of a *Prunus* pan-genome, based on already-sequenced *Prunus* genomes^143,144^. This *Prunus* pan-genome would promote and accelerate the development of appropriate genomic tools for all *Prunus* species, including wild relatives.

Whereas all mentioned resistance studies deal with one single pest or pathogen at a time, fruit trees are challenged in the orchard by a cohort of pests and pathogens, in combination or successively. Moreover, plants in the field are under constant threat of multiple abiotic and biotic stresses. Very complex physiological and molecular interactions take place, resulting in unique response(s) of the plants to withstand the combined effect of these stresses. In such situations, plants can show either enhanced or reduced pest/pathogen resistance. While the combined effect of biotic and abiotic stresses has been extensively studied in annual crops, only one study dealt with a perennial host (*Populus* sp.) and none on fruit trees^145^. This establishes a need for studies combining resistance mechanisms to multiple pests and pathogens as well as resistance/tolerance to both biotic and abiotic stresses. Hence, future research programs should explore the utilization of association mapping approaches based on high-throughput genotyping of stone fruit, wild and crop, germplasm collections that will be tested for a variety of biotic and abiotic factors.

Today, epigenetics has become a crucial research field in plant responses to biotic and abiotic stresses, especially in long-lived species such as fruit and forest trees. Plants subjected to contrasting environments have evolved different epigenomes, suggesting that epigenomes are remodeled in plants under stress, such as pathogen and herbivory attacks^146,147^ contributing to long-term adaptation^34^. Recently, several studies provided valuable information related to the epigenetic control in plant stress adaptation. Epigenetics refers to the variability in gene expression occurring as a result of modification of DNA and associated proteins. Hence, epimutations are often associated with specific sequence contexts, including tandem and inverted repeat or TE insertions that are thought to attract DNA methylation to nearby loci^34,148^. This feature, together with comparative epigenomic studies, may provide an interesting way to screen for new epialleles associated with responses to biotic stress^149^. However, such whole-genome surveys of epigenome variation are seriously missing in stone fruit tree species research. We need pilot experiments conducted by a coordinated action of laboratories that combine whole-genome bisulfite sequencing, ChIP-seq, and transcriptome analysis in order to successfully map epigenetic factors controlling responses to biotic/abiotic stresses and, in consequence, identify new phenotypic diversity.

### Tree architecture

Tree architecture can have a substantial impact on yield and management costs and also is of interest to peach ornamental and rootstock breeding programs. Different peach tree architectures have been described including the standard spreading form, brachytic dwarf, semi-dwarf, compact, spur-type, narrow-leaf, weeping, columnar and upright, or semi-columnar^150,151^.

Brachytic dwarfing in peach is controlled by a single gene, *Dw/dw*, located on chromosome 6 that was cloned using a bulk segregant analysis approach combined with genome resequencing^152^. The responsible allele is a loss-of-function mutation in a gibberellic acid receptor (*PpGID1c*) gene (Table 1). A different allele of this gene also causing brachytic dwarfism has been identified^153^. Another gene responsible for dwarfing in peach, *Tssd/tssd*, causes the temperature-sensitive semi-dwarf phenotype and has been fine mapped to a region spanning 500 kb on G3, where 69 genes are annotated in the *Prunus* reference genome^154^.

Trees with weeping habit or, by contrast, with branches with vertical growth (pillar) have been identified. For both traits, the genes causing their atypical branch growth orientation have been identified and characterized. In the case of weeping habit, the trait is determined by a single gene (*Pl*/*pl*) located on G3^118^. Using a genomic sequencing approach, a SAM domain gene (*ppa013325; Prupe.3G200700*) affecting branch orientation has been recently identified and functionally validated by silencing in *P. domestica* trees^155^ (Table 1). The trait characterized by vertically oriented branches called broomy or pillar is determined by a major gene (*Br/br*). The heterozygous phenotype has a distinctive phenotype called upright. A *PpeTAC1* candidate gene for *Br* has been identified on G2 coupling bulked segregant analysis with NGS^156^ (Table 1). This gene is a putative ortholog of rice *TAC1* (*tiller angle control 1*). The mutation causing the pillar phenotype is an insertion located in exon 3 of *PpeTAC1* (*ppa010082; Prupe.2G194000*) causing a stop codon. RNA interference (RNAi) silencing of this gene in *P. domestica* produced trees with a more extreme pillar phenotype than the one described in peach^157^.

All the available knowledge of genes controlling these traits and molecular markers to select them makes it possible to efficiently breed new cultivars with characteristics more adapted to different growing practices, including the possibility of high-density plantations and mechanization^158^. Furthermore, other traits related to root architecture need to be included in the global picture. Overexpression of one of these genes, deeper rooting 1 (*DRO1*), in *P. domestica* resulted in trees with deeper roots^159^ that could produce plants more adapted to drought.

### The gametophytic self-incompatibility (GSI) system of *Prunus*

Fertilization and seed formation are essential for fruit production in *Prunus* because stone fruit and almonds are unable to bear fruit parthenocarpically. Special attention, therefore, has been given to the *Prunus* GSI system and extensive studies have been conducted to elucidate physiological, genetic, and genomic aspects of GSI. During the past two decades, the specificity determinants of pistil (S-ribonuclease; S-RNase) and pollen (*S* haplotype-specific F-box protein; SFB) in *Prunus* have been identified by classical protemoics and genomics techniques based on two-dimensional gel electrophoresis and chromosome walking, respectively^160,161^. Identification of the pistil *S* and the pollen *S* determinants led to the development of PCR-based *S* genotyping and marker-assisted selection for self-compatible (SC) individuals. Furthermore, a series of molecular and genetic analyses of *Prunus* SC *S* haplotypes revealed the possible existence of a distinct recognition mechanism in the S-RNase-based GSI system in *Prunus*^8,160^.

Recent studies utilizing WGS information in *Prunus* shed light on the evolution of the *S* locus and establishment of the *Prunus*-specific GSI recognition mechanism. Evolutionary paths of the establishment of *Prunus S-RNase* and *SFB* were investigated by tracking their gene duplication patterns^162^. Phylogenetic analysis and estimation of proxy ages for the establishment of *S-RNase* and its homologs in several rosaceous species showed that the divergence of *S-RNase* in the subtribe Malinae and the genus *Prunus* predated the gene in the most recent common ancestors of Rosaceae species. Furthermore, the duplicated S-RNase-like genes were accompanied by duplicated pollen S-like F-box genes, suggesting segmental duplications of the *S* locus. Analysis of the expression patterns and evolutionary speed of duplicated S-RNase-like genes in *Prunus* suggested that these genes have lost the SI recognition function, resulting in a single *S* locus. Furthermore, phylogenetic analysis with SFB and its orthologs in other angiosperm genomes indicated that *Prunus SFB* does not cluster with the pollen *S* of other plants and diverged early after the establishment of the Eudicots^29^. *Prunus SFB* likely originated from a recent *Prunus*-specific gene duplication event. Transcriptomic and evolutionary analyses of the *Prunus S* paralogs are consistent with the establishment of a *Prunus*-specific SI system and the possibility of subfunctionalization differentiating the newly generated *SFB* from the original pollen *S* determinant. The *S* loci in the current Rosaceae species might have evolved independently from the duplicated *S* loci, which could explain the presence of genus-specific SI recognition mechanisms in the Rosaceae.

Recently, WGS information has been further utilized to identify pollen part modifiers specifically present in *Prunus*^63,163^. Availability of WGS information in diverse species of *Prunus* and resequencing information of cultivars and strains in a given *Prunus* species will further contribute to elucidating the *Prunus*-specific GSI system.

## Applications of genome information in *Prunus* improvement: MAB, MAI, genomic selection, and visualization of the genome-wide genetics of elite individuals

Routine application of MAB has long been the main avenue to which stone fruit breeders have expected to benefit from genomics advances. The ability to predict the phenotype before it is expressed and especially in the early stages of each plant’s life is of high value in crops that can take ≥5 years before the phenotype can be assessed in the field. Several collaborative attempts to translate that vast information of QTL and gene analysis in user-friendly informative DNA tests have been attempted^164,165^ (www.fruitbreedomics.com; www.rosbreed.org). However, only a limited number of reports on development, validation, and successful implementation of MAB have been published so far^79,87,94,102,133,166–172^. To aid in publicly sharing DNA information, the promises, progress, and prospects of using DNA-based information in breeding in Rosaceae and other horticultural crops have been recently summarized^173–175^ and steps to translate promising QTL information to a trait-predictive DNA tests have been proposed^176^. With additional *Prunus* genome sequences being released, an exponential increase in discovery of marker–trait associations is expected. A shift in selection approaches to molecular genotyping and DNA informed breeding efforts have proven useful and are now economically feasible on much larger scales.

The variability of wild and cultivated compatible relatives of *Prunus* species is an enormous reservoir that may contain useful alleles to be integrated into target species, particularly those that have less diversity such as the peach^20,177^. However, this variability has seldom been used in *Prunus* breeding because of: (1) poor knowledge of the inheritance of traits of interest that could be introgressed from exotic sources; (2) limited availability of interspecific crosses and incomplete knowledge of intercrossability among species, and (3) the several generations required to recover an elite genetic background to result in commercially valuable fruit tree cultivars. A method to address these drawbacks, MAI, was proposed and tested successfully in peach × almond crosses^142^. MAI consists of generating a large BC1 population (*N* > 1000) from an interspecific hybrid. A small collection of seedlings (15–25) with a few introgressions (the “prIL set”) and overall containing the whole donor genome can be selected from this progeny. These individuals, when backcrossed again or selfed, will produce with reasonable frequency genotypes with the genetic background of the recurrent species, peach in this case, and a single fragment in homozygosis or heterozygosis of the donor parent (almond). In the case of the peach × almond progeny used, the first individuals with a single introgression were extracted 9 years after the beginning of the experiment^142^. Currently, a collection of introgression lines (ILs) heterozygous or homozygous for a single introgressed almond fragment is available with 85% and 45% coverage of the almond genome, respectively (P. Arús unpublished results). Phenotyping the prIL set is also an opportunity for identifying, with low resolution, the genome positions of major genes with dominant or additive almond alleles. The characters of interest can be examined in a genetic background very close to that of the recurrent parent and therefore with limited influence of the donor variability at other genetic regions. This situation makes the prIL set an interesting tool for assessment of the variability supplied by an exotic genome and is an affordable means for long-term storage of the exotic genome in an elite background, often only one generation away from a commercial cultivar. Implementing MAI approaches to other crop × wild or exotic *Prunus* species is an interesting objective for ensuring future availability of the overall diversity of this genus for breeding purposes.

Genomic selection is a molecular genotyping technique that shows promise in fruit tree crops for enhancing breeding efficiency via increased prediction accuracy and selection intensity and decreased generational interval^178–180^. Application of genomic selection in plant breeding is still fairly new and requires further investigation of the most appropriate model(s) and the optimal number of markers and plants to use^181,182^. Preliminary reports on the factors affecting accuracy of genomic prediction in fruit crops suggested importance of relatedness between the reference and validation individuals, with prediction accuracy increasing in highly related material^178,180^. In addition, multicycle analysis in strawberry^180^ and peach^179^ showed a steady increase of prediction accuracy for all traits as data were aggregated across cycles in the reference population.

Genetic architecture of the trait influences the size of the reference population needed to accurately estimate the SNP effects. The smaller the largest SNP effects are, the larger a reference population is needed^178^. Modeling for the optimal number of markers in apricot^61^ and strawberry^180^ suggested that prediction accuracy of 88–97% is achieved with ~500–1000 informative, non-redundant, randomly distributed markers. Evaluation of the feasibility of genomic selection in peach for highly polygenic traits linked to yield and fruit quality suggested similar prediction accuracy with over 60% for fruit weight, sugar content, and titratable acidity^179^.

Genomic information can also be used to estimate genetic relationships among germplasm populations by modeling the performance of an individual in different environments^183,184^ and to estimate an individuals’ breeding value^49,179,184,185^. High correlation (0.88; *P* < 0.0001) between predicted genomic breeding value and fruit size was observed in cherry^185^. An initial study on predicting genomic breeding value for fruit weight, sugar content, and titratable acidity in peach showed promising results for application of genomic selection in peach breeding programs^179^.

**Fig. 3:**
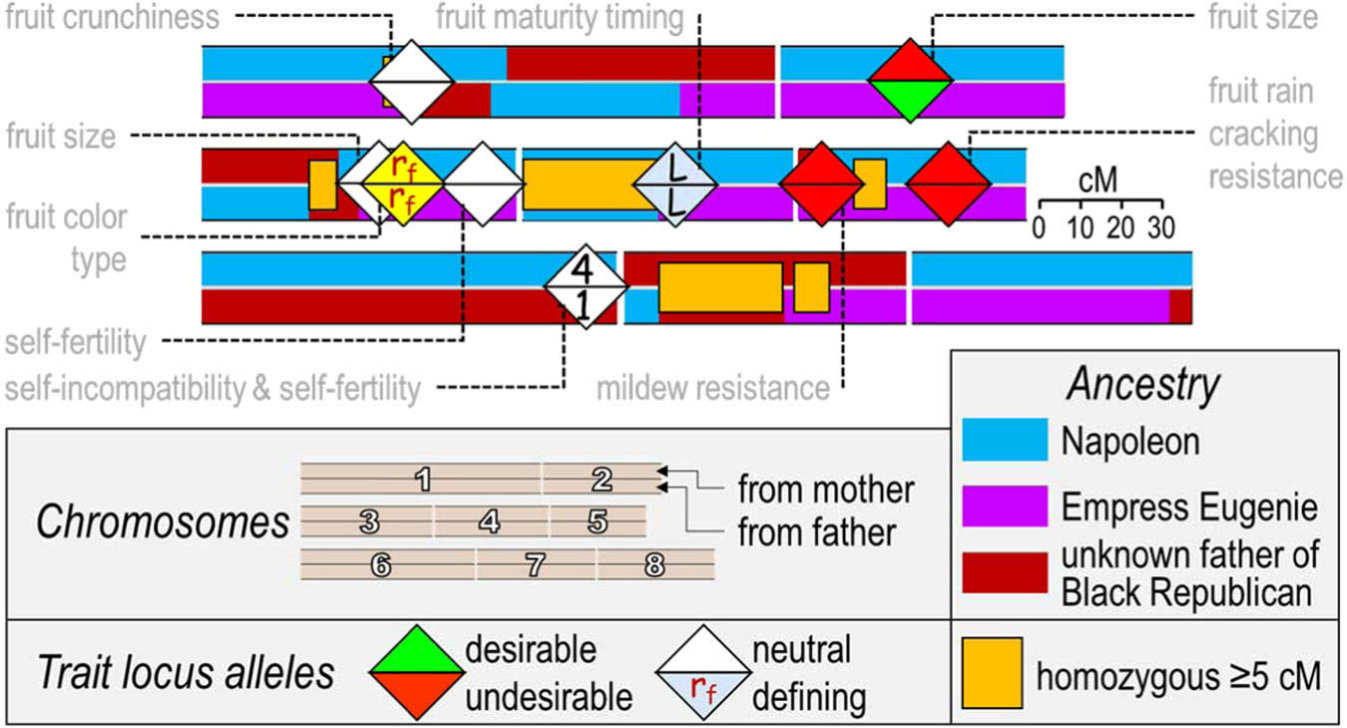
Haplotype mosaic of “Rainier”, a classic Washington-bred sweet cherry cultivar. Segments that “Rainier” inherited via its parents from its three specified ancestors are displayed across the eight chromosomes of sweet cherry. In some cases, these ancestral segments are homozygous, highlighting consequences of inbreeding and signifying common ancestry in generations behind known ancestors. Trait locus alleles are indicated with phenotypic effects and ancestral origins; despite the commercial success of “Rainier,” it can be seen that there is still much to be improved. These results were obtained from single-nucleotide polymorphism (SNP) data curation and pedigree ascertainment by L. Cai and C. Peace using the RosBREED cherry 6K SNP array v1 on a U.S. breeding germplasm set (*n* ~ 500)^21^. Diagram is from Peace et al.^175^

A new opportunity is available for breeders to effectively access and make decisions on the genetics of their germplasm. The breakthrough has four components. The first is a technological advance in genotyping technologies that efficiently provide genome-wide genetic polymorphism data; SNP arrays are particularly useful here because the same loci are assessed on all plants. The second component is conceptual, giving explicit attention to each elite germplasm individual (parents, selections, and cultivars) just as breeders do. The common way to deal with large genotypic datasets is a quantitative summary across the germplasm, but this approach easily overlooks each germplasm individual. The third component is an analytical synthesis of genome-wide genotypic data into three genetic vectors of breeding relevance: (1) allelic variation (patterns of similarity among alleles within each locus); (2) linkage (patterns of recombination among loci within each individual); and (3) relatedness (patterns of shared ancestry among individuals within a population). Together, the patterns of alleles over loci over generations can provide emergent genetic knowledge on elite germplasm individuals to improve the precision with which individuals are used in breeding. The key qualitative leap in information now available is the positioning of all recombination events that have led to each plant. SNP datasets of pedigree-connected material must be carefully curated by removing errors, ascertaining all pedigree connections, phasing each individual, and imputing missing data^186^. Haploblocking (section “Haploblocking *Prunus* genomes”) also helps. The fourth component is enabling user experience of the holistic “genotype” of each elite individual. It is proposed that sensory experience of DNA information that focuses on elite individuals, just as for phenotypic information, would help breeders better understand what they have and then target development of what they want. While DNA-based genetic information cannot yet be heard or tasted, one way for breeders to “experience the genotype” of an elite individual is by seeing it. Visual aids are well recognized as vital tools for transmitting information for long-term memory retrieval and empowering users to leverage their creativity and understand large data patterns^187^. While improvements in graphical genotyping software are desired as the fifth component to facilitate routine use of genome-wide genetic information in breeding decisions, in the meantime ad hoc collation of the above information can be made as “haplotype mosaics” (Fig. 3). Software is in development to display a circular format for haplotype mosaic visualization. In sweet cherry, visualizing the genetics of elite genomes has recently been used to reveal new pedigree relationships among cultivars and trace origins and distributions of valuable trait locus alleles in ancestors and parents^188^.

## Development of biotechnology tools: genetic transformation and gene editing

The lack of efficient regeneration systems in *Prunus* is a primary limitation for stable genetic transformation procedures and generation of transformant individuals. New breeding techniques (NBTs), involving approaches such as RNAi, DNA methylation, and gene editing, could overcome the monolithic requirement of having a final T-DNA-modified individual. Novel techniques in gene transfer have a rediscovered value due to simple requirements such as an efficient transient expression^189^ or a direct intake of pre-assembled^190^ editing reagents into the cell. For this reason, transient expression systems, protoplast regeneration, somatic embryogenesis, and organogenesis regeneration procedures are currently being optimized for NBT application.

The availability of genome drafts in *Prunus* species enables advances toward improved and safer applications involving NBTs. Regarding gene editing technology, prior knowledge of the genome can help to ensure system efficiency and specificity. The Clustered Regularly Interspaced Short Palindromic Repeat (CRISPR)/*Cas*9 system, discovered as an adaptive line of defense against viral infection in *Archaea*^191^, is the most common gene editing technique that allows for the direct generation of sequence modifications in the genome. Targeted mutagenesis by this tool involves making guide RNAs (gRNAs) that target customized sequences in the genome to direct the *Cas*9 nuclease activity to generate double-strand breaks adjacent to the gRNA-joined location. gRNA refers to a short synthetic RNA composed of a scaffold sequence necessary for *Cas*-binding and a user-defined 20 nucleotide spacer that determines the genomic target to be modified. The target sequence recognized by the spacer will be a protospacer sequence, located contiguous to an adjacent motif recognized by *Cas*9 required for DNA cleavage, that is an NGG nucleotide arrangement called protospacer adjacent motif (PAM). Currently, new nucleases replacing Cas9 are the focus of new research that uses different PAM motifs^192^.

By computing on described genomes, PAM datasets and their contiguous regions can be reviewed and summarized in datasets. In this way, several datasets for gRNA and PAM required in DNA editing have been described and used for on-target and off-target predicting activity of gRNAs inside a genome^193^. In the case of *Prunus* spp., *P. mume* datasets with information about the target sites of Cas9 (i.e., NGG) and newer nucleases such as Cpf1 (which uses TTTNs and TTNs as PAMs) are available at the CRISPR-Local^194^ website (http://crispr.hzau.edu.cn/cgi-bin/CRISPR-Local/download). Similarly, based on the peach and sweet cherry genomes, a Cas9 gRNA designer and analyzer for both species is available (www.fruit-tree-genomics.com/biotools).

The system CRISPR/Cas9 has been successfully used in plants and delivery of editing components into the plant cell has been mostly achieved by their stable integration into the genome by gene transfer techniques relying on *Agrobacterium*-mediated transformation^195^. Efforts to edit individuals without foreign DNA insertion into the genome have involved the delivery of assembled ribonucleoprotein editing reagents^190^. A different approach implies the delivery of DNA-replicons using disarmed or “deconstructed” viruses^189^, which allows for a high copy number in the cell without the insertion of the replicon into the plant genome.

Efficacy of the above-mentioned techniques in vivo relies on the knowledge of the specific target sequences and, ideally, of whole-genome information to prevent secondary effects on eventual off-targets. For this reason, transient expression assays have become relevant as screening systems prior to a “precise breeding” full-path experimentation. These procedures are relevant because they avoid the technical difficulty and the time needed for whole plant generation, which is a major restriction in *Prunus*^9,196^. Protocols based on the use of explants such as leaf discs, stem segments, or even whole plants have been achieved in other woody species such as grapevines^197,198^. Nevertheless, these approaches are restricted to a few *Prunus* species such as *P. domestica*^199^ and *P. salicina*^200^ in experiments that can be considered preliminary assays that later led to stable transformation procedures^201,202^. In *P. persica*, different tissues were successfully used as explants for biolistic-mediated β-glucuronidase gene expression^203^ in the search for a stable transformation procedure. In addition, protoplast procedures in *P. avium*^204^ and *P. avium* hybrids^205^ can be proposed as optimal tools for gene editing capability for in vivo evaluation. This technique could help to overcome chimerism, a significant issue in woody plant genetic transformation.

A significant amount of research has been carried out regarding *Prunus* spp. regeneration using different explants and approaches, and optimal conditions for this process are determined by many factors^9^. Whereas leaf explants can lead to adequate regeneration systems in several members of the Rosaceae family^206^, extremely recalcitrant species in *Prunus* limit regeneration mostly to using seed explants^9^. Recently, leaf explants of *P. domestica* have shown increased adventitious shoot production by constitutive expression of the class I *KNOX* gene from corn^207^.

Improved regeneration efficiencies have been obtained in other species. In sweet cherry varieties, more efficient methods were described using leaf and nodal segments^208^ and mature cotyledons^209^. Better results have been reported in other commercially important genotypes, including sour cherry^210,211^, black cherry (*P. serotina*)^212^, and several cherry rootstocks^211,213,214^. In another example, a complete pipeline of methods for transformation and regeneration of transgenic apricot plants from “Helena” leaves has been described^215,216^. In all cases, researchers used *A. tumefaciens* EHA 105 for gene transfer.

Experimental procedures exploiting RNAi approaches allowed the generation of *Prunus necrotic ringspot virus* (PNRSV) resistant individuals of a cherry rootstock^217^. Grafting of these individuals demonstrated small RNA transport from rootstock to scion, resulting in “Emperor Francis” scions becoming resistant to PNRSV^218^. In this way, the use of *Prunus* genotypes currently suitable for genetic transformation can be proposed as small RNA generators (donors) for systemic transmission of resistance through transgrafting to compatible scions from different genetic backgrounds, directing either systemic sRNA-directed gene silencing or sRNA-directed DNA methylation.

## Existing and new bioinformatics tools and resources for *Prunus*

The availability of extensive sequence data forms an essential genomics resource in designing various research platforms to understand the biology of crops and to apply the knowledge in their improvement. Three primary sequence databases are GenBank^219^, European Nucleotide Archive^220^, and the DNA Data Bank of Japan^221^. As of September 12, 2018, a search of NCBI for data on *Prunus* revealed 4 genomes, 85,014 genes, 196,367 proteins, 2,994,795 nucleotide sequences, 2481 probes, and 2712 records in the short read archive (SRA). The WGS data include *P. persica* v2.0^10,16^, *P. avium* v1.0^12^, *P. mume* v1.0^11^, and *P. yedoensis* var. *nudiflora* (assembly Pyn.v1)^13^. Users can also download the WGS data from their FTP site and perform BLAST analyses. In the GDR^222^, in addition to two assemblies of peach, *P. persica* genome v1.0 (IPGI 2013) and v2.0, the whole-genome assembly of sweet cherry *P. avium* genome v1.0 and almond *P. dulcis* Texas v2.0 are available for *Prunus*. Additional data provided by GDR on these assemblies include computational annotation of predicted genes with homology to genes of closely related or model plant species and assignment of InterPro protein domains^223^ and GO terms^224,225^. NCBI runs a separate gene annotation pipeline to annotate WGS data. To help researchers compare two different gene annotation sets, GDR performs BLAST analysis between the NCBI annotated genes and genes from the original genome assemblies. The WGS data in GDR can be accessed through the species page, gene/transcript search page, JBrowse^226^, and BLASTX^227^. Newly added functionality in the gene/transcript search page allows users customize output to include various functional annotation data in the result table.

The *Prunus* SNP array data and the genotyping data using these SNPs are available to view, search, and download in JBrowse and other pages in GDR. In the SSR and SNP genotype search pages, data from nine SSR and four SNP genotyping projects are available for *Prunus*. The new SNP genotype search page as well as a new plugin in JBrowse in GDR allows users to choose a specific genomic region and peach accessions to view the individual genotype.

The latest and most efficient tool for transcriptome analysis, RNA-seq, allows not only the assessment of the expression level of specific genes but also the detection of less-represented transcripts, allelic-specific expression of transcripts, post-transcriptional mutations, and the expression of splice variants. GDR provides a page that links to *Prunus* RNA-Seq and DNA datasets in the NCBI SRA. The expression data from selected RNA-seq analyses will be available from GDR using Tripal Analysis Expression Module^228^. This module allows users to submit gene sets to generate a heatmap and also view expression patterns of a mRNA in a feature page. The published RNA-Seq and dbEST datasets are also analyzed by GDR to create a reference transcriptome (RefTrans) for major species and provides putative gene function identified by homology to known proteins. For *Prunus*, *P. avium* GDR RefTrans v1.0 and *Prunus persica* GDR RefTrans v1.0, are currently available. Currently, there is not much epigenomic data available for Rosaceae but more data are expected to come^229^ and search and analysis tools for epigenomic data will be available in GDR.

With the increasing number of species with WGS, several web-based databases are available for comparative genomics. A few contain the WGS of Rosaceae species. CoGe (The Place to Compare Genomes)^230^ contains data of 48,063 genomes including most of the Rosaceae genomes. Plaza^231^ and the plant genome duplication database (PGDD)^232^ contain some of the Rosaceae WGS, including *P. persica* v1.0. Phytozome^233^ currently contains *P. persica* v2.0. In GDR, *P. persica* v2.0 is used in a synteny analysis with eight other Rosaceae whole-genome assemblies using MCScanX^234^. The synteny results are available through the new Synteny Viewer. The gene/mRNA page also lists the orthologs and paralogs identified in this analysis and provides a link to the Synteny Viewer.

One of the most important roles of GDR as the community database for the Rosaceae family is to integrate all the different types of data to maximize the value. In addition to the data mentioned above, GDR includes genetic data, such as map; marker; and QTL, genotype, and phenotype data. GDR currently contains 168 genetic maps for *Prunus*, which can be viewed and compared through a new graphic interface, MapViewer. Trait locus data for *Prunus* in GDR includes 1491 QTLs and 39 major genes for 148 horticultural traits. GDR contains 471,854 *Prunus* genetic markers including 151,479 SNPs. The SNP data are available as JBrowse tracks, downloadable files, and to search and download from the SNP marker search page as well as the all marker search page. The marker search page has a new feature, filtering by trait name, allowing users to search for markers that are associated with QTLs. In addition to the genotype data, phenotypic data from projects such as RosBREED, are available through the “Search Trait Evaluation” page. The public breeding data can also be accessed using the Breeding Information Management System (BIMS). BIMS allows Rosaceae breeders to store, manage, archive, and analyze their private or public breeding data. Future efforts in GDR include more data curation, integration, standardization, and further tool development to utilize the integrated data across crops, data types, and discipline.

## Conclusions

*Prunus* encompasses a group of economically important and closely related crops, sharing an essentially common genome. Each of them followed a distinct pathway of evolution pre- and post-domestication leading to various biological and reproductive outcomes, including that certain species remain intercrossable producing fertile progeny, while others are reproductively isolated. New sequence-based approaches provide an opportunity for the in-depth study of the genetics and evolution of this unique group of crops. Evolutionary studies of other perennial crops based on whole-genome sequence data are already underway, such as in apple^235^ and grape^236^. These studies have uncovered interesting patterns of evolution during and after domestication, including identification of selective sweeps, evolution of population sizes, and introgression from other species as part of the domestication process. Overall, perennial crop evolution appears to follow specific trends as compared with the more extensively studied annual crops^237^. Similar studies for *Prunus* are starting to emerge, particularly in peach^20,25,27,29^ and almond^26^. These results, including the comparison of evolution patterns in a group of closely related crop species, are likely to provide fascinating stories that will help to understand the nature of available variability and its potential for developing fruit crops needed for the future.

Even with synteny between *Prunus* species so high, having a single high-quality peach sequence is insufficient. The genomes of some other *Prunus* species have been recently released or will be in the near future, but there is still a need for extending the de novo sequence to all crop species of the genus, a set of key wild relatives and several representatives for each crop selected to cover their main variability/population structure transects. The interspecific and intraspecific variability that constitutes the pan-genome of *Prunus* has to be characterized. This is crucial for understanding the causal mechanisms explaining the existing genetic diversity among cultivated species and for the analysis of chromosomal regions containing the genes responsible for the variability of important traits within each species.

One of the features of *Prunus* variability is the existence of a large set of genes with major effects on the phenotype (major genes or major QTLs)^4,238^. This is particularly important in the case of peach and may have been favored by its self-compatibility system, enabling for the easy generation of selfed progenies with low or no inbreeding depression, and by artificial selection in the cultivated species for obvious mutants in the fruit, flower, plant habit, and many other characters. Identifying the genes controlling these traits and characterizing their molecular variability has important basic and applied consequences. The progress realized since the release of the first peach genome on gene cloning has been enormous (Table 1) despite inherent difficulties of long intergeneration time, low seed production and germination, and the recalcitrant in vitro behavior of most *Prunus* species. This trend will likely continue in the next decades, further enabled by the exponential increase of DNA sequence information availability and progress of gene editing approaches. These technological advances may help to circumvent some of the current limitations for functional validation of candidate genes and incorporating new valuable alleles into new cultivars of these species.

Establishing routine MAS for major genes in breeding *Prunus* and other temperate fruit crops^117,174,175^ is a first step toward employing whole-genome approaches in selecting better cultivars. Knowledge of the genotype across the genome in large sets of historical and modern accessions enables the establishment of highly reliable pedigrees and ways of visualizing, and thus better understanding and using, the available variability in the set of parents used in each breeding program. In addition, this approach allows the complexity of the genome to be simplified by using concepts like haploblocking. Whole-genome selection with a set of markers loosely covering the genome greatly reduces the number of generations needed for recovery of elite germplasm after an elite × wild or exotic cross as demonstrated by MAI^142^. This opens the door for understanding and capturing the genetic variability present in the enormous gene reservoir of the *Prunus* wild and cultivated species. Similar approaches can be used to identify individuals highly similar to a given high-value heterozygous genotype in its selfed progeny or to select two complementary homozygotes that, when crossed, produce a genotype similar to one of contrasted value as described in “resynthesis”^239^. Combined with MAI, this strategy provides a way of introgressing new alleles of interest into an established heterozygous genotype. Finally, genomic selection is a powerful approach for *Prunus* crops with their long intergeneration periods, and research so far shows that it is feasible and potentially useful^61,179^. It is now crucial to design and test genomic selection schemes specifically adapted to the needs of perennial crops. Overall, the increasing amount of DNA information on genes and QTLs and the development of new breeding strategies and methods such as gene editing are providing a significantly enhanced toolbox that can provide a strong push to the development of superior new cultivars. The use of this enhanced toolbox and its integration into the public and private breeding programs will require additional investments in training and translational research^175^, following in the steps and leveraging the achievements of successful large-scale European^164^ and US^165^ initiatives.
